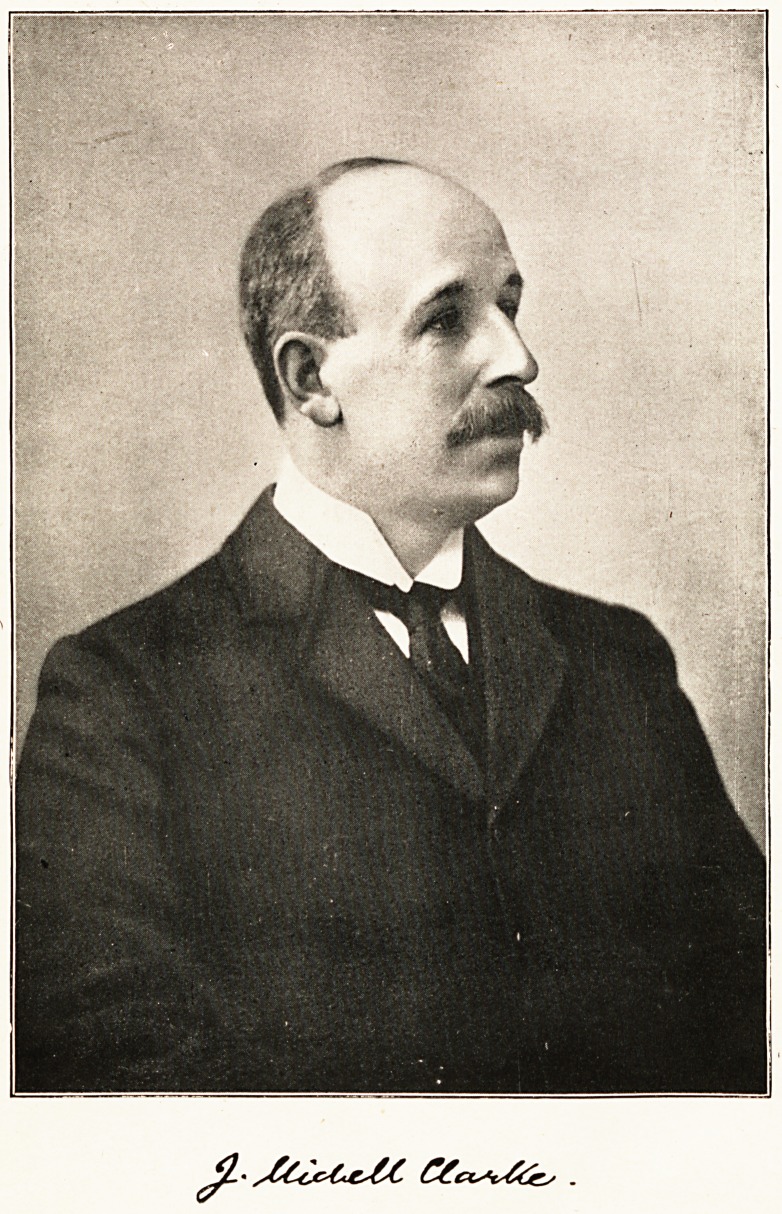# John Michell Clarke

**Published:** 1918

**Authors:** 


J. Michell Clarke.

### Obituary.

---

**JOHN MICHELL CLARKE, LIEUT.-COL. R.A.M.C.T., M.A., M.D., F.R.C.P., LL.D. BRISTOL.**

THE loss of our good friend and colleague, John Michell Clarke, will arouse the deepest regret in our readers, who have so often enjoyed articles from his pen. For over twenty-five years he had taken an active part in the Editorial management and the laborious task of revising and correcting the material for each quarter's issue.

His brilliant scientific achievements, combined with an excellent judgment, and his quiet, modest demeanour, are known to all; but his fellow-workers realise sadly in looking back his remarkable energy and boundless capacity for work. Nothing strikes one more now than the great number of public duties which he carried out efficiently, unless it be the good temper and tact with which he performed them. His whole life was one constant devotion to duty.

It may be interesting to recall that he was the son of a highly esteemed and successful medical man, Mr. W. Michell Clarke, who preceded him on the Staff of the General Hospital, and who was, like his son in later days, President of the Medico-Chirurgical Society and of the Branch of the British Medical Association.

John Michell Clarke was born at Clifton in 1859, and was sent first to a preparatory school (Miss Leedham's), and then to Dr. Hudson's, of Manilla Hall, one of the famous private schools which flourished here at that time. When he was sixteen, he passed into the new and stately buildings of Clifton College, which had recently commenced its prosperous career. Here for three years as a South Town boy he worked his way to the Sixth Form, and left to enter at Gonville and Caius College, Cambridge.

The medical school there had recently been reorganised under Sir George M. Humphry, one of the most inspiring teachers of the age, who gathered around him such brilliant men as Michael Foster, Gaskell, and Balfour in a determined effort to make that school the most progressive and scientific in England. Clarke soon absorbed the enthusiasm and spirit of his surroundings, but after taking his degree in the Natural Sciences Tripos of 1882 and holding a Demonstratorship of Anatomy he had to leave for clinical study elsewhere.

He dressed for Mr. Nelson Dobson at the Bristol General Hospital, but had the misfortune to get diphtheria in an attempt to revive a drowning man. For a long while he was ill with cardiac complications, but eventually regained vigorous health and went up to St. Thomas's Hospital, where he made the most of his opportunities of studying medicine under such teachers as Ord, Bristowe and Buzzard. He was holding office as House Physician when the sudden death of his father in 1885 and the need of taking his place in Clifton forced him to give up his appointment and the possibility of practice in London.

Settling down in Clifton, he was elected Assistant Physician and Pathologist at the Bristol General Hospital in 1886, and from that time to the day of his death nothing ever lessened the devotion with which he worked for that hospital and his patients there. He was made full Physician in 1893, and for thirty years he took an active share in all the improvements made during that period. In his earlier days he did most of the pathological and museum duties himself, but later on the Committee put up a fine range of buildings and appointed a whole time Pathologist in response to the representations of Clarke and his colleagues. In this as in every other matter his time and careful thought were ungrudgingly given both to the Committee and the Staff, by all of whom his sterling qualities were most warmly appreciated. At this time a movement was going on in the country for the better training of nurses and the improvement of their status. Michell Clarke gave himself warmly to support the efforts of the Hospital Committee, and changes were worked out which revolutionised the education of nurses there, enabling them to make some provision for the future, and created a highly-trained staff, who were available both for the Hospital and for private cases. He felt keenly that the Hospital should be a school for the best possible education in nursing, and it was largely due to his constant co-operation that the Committee realised many of their ideals. He joined, too, in the work of a Society in Clifton with similar aims, which started and organised the first great Nursing Home with its staff of trained nurses, and is still carrying on a successful work in Chesterfield Place.

At the Bristol General Hospital he found one of his greatest interests in his duties as Dean for the medical students. His inspiring and methodical teaching drew to him a crowd of pupils, to whom he endeared himself by his constant care for their interests as well as by his scientific acumen and powers of exposition.

At the time he began professional life he saw that the opportunities for an advance in Neurology were very great. Hughlings Jackson, Ferrier, Gowers, and some Continental workers had opened up new methods of study. He threw himself with all his vigour into the localisation and pathological anatomy of nerve diseases, studying and utilising every case he met with, and spending his leisure hours in laboratory work. In this field his research and observations produced scientific gains of permanent value, some of which he found time to publish in the sixty neurological papers given with his other writings in the list below; but too many, alas! remain buried in the carefully written note-books he accumulated. His studies on hysteria, on cord degeneration, and on the less known forms of paralyses are of great importance, but his devotion to Neurology did not prevent him from throwing new light on other medical subjects which came before him. Thus we owe to him a careful research on Trench Nephritis, which he saw among the soldiers at Southmead.

The Bristol Medical School, after more or less disorganisation during the eighteenth century, had been definitely revived some sixty years before Michell Clarke began to practise. It had been united to the new University College, but though vigorous it suffered under great disadvantages. The first of these was the impossibility of giving any degree or registrable qualification. There was also an almost entire absence of any endowments and of whole time teachers. One of the chief teachers complained that the dividend available for his course of lectures only amounted to eighteenpence. Still active teaching was carried on and a good average of successes in the London examinations was maintained. Michell Clarke lectured first in Physiology and then in Pathology, and as usual inspired his students with his own enthusiasm and scientific feeling.

An immense amount had to be done as funds came in to organise the work of the School more thoroughly. In the endless negotiations which followed and were continued when the Charter of the new University was obtained Michell Clarke's solid judgment and his skill in harmonising conflicting interests were of the utmost value. To a remarkable capacity for taking pains he joined a quiet but forcible way of stating his views, which were generally felt to be reasonable and always straightforward and disinterested.

When the University was an accomplished fact he became one of the Professors of Medicine, then Pro-Vice-Chancellor, member of the Council and of the Senate, and for a time Chairman of the Board of the Medical Faculty, all of which made further inroads on his time. His abilities had already been recognised by the Fellowship of the Royal College of Physicians (London) in 1896, and in 1917 he was placed on the Council of that College. Cambridge, too, had recognised his worth by making him an Examiner for the M.B. degree.

On the other hand, he had toiled for ten years for his brethren as Secretary of the Branch of the British Medical Association, with its arduous and often delicate duties. Then to help the Panel practitioners he served for other years on the Local Insurance Committee, when local feeling was running high; and unlike many men with a quarter of his work he gave a steady attendance at the scientific meetings of our Society and those of the British Medical Association, and contributed largely to the discussions.

His addresses as President of both Societies were characterised by their solid value and their happy phraseology. The list below shows the large number of contributions he made to our *Journal*, and he was also an Editor of the *Quarterly Journal of Medicine*, of *Brain*, and the *Journal of Pathology*.

He gave active help, too, in assisting at the foundation of the Winsley Sanatorium, and to the Research Defence Society. As Consulting Physician to the Clifton Dispensary and a Governor of the Victoria Convalescent Home he found other fields of usefulness.

With all this he carried on a large consulting practice, which entailed long journeys over the West of England, as his scientific reputation and character were widely recognised.

When the war broke out he was at once called up as an *à la suite* officer and attached to the Southmead section of the Second Southern Hospital as Senior Physician, with the extra work of the Electrical Department. Summer and winter he covered the three-mile journey by 9 a.m., and before long as Lieut.-Col. he was placed in charge of the administration of the entire section, which was raised to 1,020 beds. This would be a heavy task of itself for one man, since he had besides the soldiers a large number of officers undergoing treatment there, and a numerous staff, among whom he succeeded in maintaining harmonious relations. However, he found time to continue his researches and notes upon his cases, and as one of the Sisters said, "he was never too busy to hear anything we had to say." After many hours at Southmead he had to travel to Bedminster for his patients and lectures at the civil hospital there, and finally to find time and strength for his private patients and literary work.

For over three years this double strain went on. Meanwhile he was called to serve on the Local and on the Central War Committees, with the anxious and trying duty of selecting those doctors who could be best spared for the Front. On the whole, the Committees succeeded in supplying the nation with a huge body of qualified men with the least possible disturbance of medical service for civilians; but it must have told heavily on a man so kind-hearted and sympathetic towards all his medical brethren.

Chance words at times showed how deeply the fluctuations of the war weighed on his patriotic feelings, but the grievous loss of his second son, who was killed at the Front, was borne with his usual quiet self-restraint.

We have not space to record his successes in golf, which he pursued with his usual thoroughness, rarely satisfying himself with his best performances, nor to describe his enjoyment of the social evenings with the century-old Medical Reading Society, where his huge appetite for new books was sometimes found to be a little embarrassing.

His scientific writings show his powers of observation and his indefatigable labour for the advancement of medicine. but it is to Michell Clarke, the man himself, to whom we wish to pay our tribute here. A man in all things straight, who never stooped to adopt a devious course to obtain his end. To his friends loyal, and to those against whom he might find himself, for the moment, opposed, he was just. Looking back, we can remember no instance where he could be suspected of being unfairly biased by self-interest. A very active and busy man, he was never too busy to help anyone who came to him in a difficulty ; he never spared himself in pains or time to right an injustice. His help was never showy, but it was admirably effective. It was a keen pleasure to him if he could himself slip away from the credit and thanks after matters were righted, but it has been astonishing to learn afterwards from the recipients what tactful and at the same time substantial help he gave to those in adversity.

### BIBLIOGRAPHY.

" On Graves's Disease with Case," *Bris. Med.-Chir. J.*, 1887, v. 17.

" Peripheral Neuritis, *Ibid.*, p. 73.

" Notes on Out-patient Cases," *Ibid.*, 1888, vi. 120.

" Progressive Muscular Atrophy with Recovery," *Ibid.*, p. 195.

" B-naphthol in Enteric Fever," *Practitioner*, 1888, xli. 421.

" Three Cases of Friedreich's Disease," *Lancet*, 1889, i. 570.

" A Case of Tetany," *Illustr. M. News*, 1889, iii. 148.

" Treatment of Locomotor Ataxy by Suspension," *Practitioner*, 1889, xliii. 339.

"Clinical Cases," *Bris. Med.-Chir. J.*, 1889, vii. 258.
"Dilatation of the Stomach," *Ibid.*, p. 21.
"Tubercle of Medulla Oblongata," *Ibid.*, p. 194.
"On some Varieties of Paraplegia," *Ibid.*, 1890, viii. 226.
"Case of Syphilitic Growth in Dura Mater," *Lancet*, 1890, i. 460.
"Remarks on Biliary and Hypertrophic Cirrhosis," *Brit. M. J.*, 1890, i. 1000.
(With J. G. Smith) "Case of Removal of Vermiform Appendix with Recurrent Attacks of Inflammation," *Lancet*, 1890, i. 956.
"Hydronaphthol in the Treatment of Enteric Fever and of Diarrhœa," *Practitioner*, 1890, xlv. 1.
"Case of Locomotor Ataxy," *Brain*, 1890, xiii. 363.
"Case of Ataxic Paraplegia with Autopsy," *Ibid.*, pp. 356, 373.
"Some Cases of Hysteria in the Male Subject," *Lancet*, 1890, ii. 1322.
"Value of Suspension in the Treatment of Tabes Dorsalis," *Ibid.*, 1891, ii. 114.
"Case of Tabes Dorsalis," *Brain*, 1891, xiv. 105.
"Case of Total Transverse Lesion in the Upper Dorsal Region of the Cord," *St. Thomas's Hosp. Rep.*, 1891, xix. 23.
"Three Cases of Intracranial Tumour," *Brit. M. J.*, 1891, i. 1283, 1334.
"Three Cases of Hysteria in Man," *Brain*, 1891, xiv. 523.
"Modern Methods of Diagnosis in Gastric Affections," *Bris. Med.-Chir. J.*, 1891, ix. 73.
"On Hysteria," *Brain*, 1892, xv. 522.
"Clinical Observations on Hysteria," *Lancet*, 1893, i. 1123, 1185.
"Multiple Abscess of the Liver," *Practitioner*, 1893, li. 270.
"Case of Intracranial Hydatid Tumour with Hemiplegia," *Brain*, 1893, xvi. 425.
"On Syphilitic Affections of the Spinal Cord," *Lancet*, 1894, i. 1297.
"Remarks on Cirrhosis of the Liver in Children," *Brit. M. J.*, 1894, i. 1407.
"Case of Friedreich's Disease," *Ibid.*, 1894, ii. 1294.
"Hysteria and Neurasthenia," *Brain*, 1894, xvii. 119.
"Left Hemiplegia and Motor Aphasia without Coarse Brain Lesions, *Lancet*, 1894, ii. 253.
"On the Pulsus Bisferiens of Aortic Regurgitation," *Ibid.*, p. 1529.
"Somnambulism," *Bris. Med.-Chir. J.*, 1894, xii. 81.
"Diagnosis of Cancer and Simple Dilatation of the Stomach," *Ibid.* 1895, xiii. 181.
"On Endothelioma of the Spinal Dura Mater," *Brain*, 1895, xviii. 256.
"Clinical Lecture on Apoplexy," *Clin. J.*, 1895, vii. 135.
"Case of Varicose Aneurysm of Ascending Aorta," *Lancet*, 1896, i. 155.
"Cases of Biliary Colic cured by Olive Oil," *Brit. M. J.*, 1895, ii. 76.
(With C. A. Morton) "Case Presenting Symptoms of Cerebral Tumour, in which a large area of the skull was removed for relief of intracranial pressure," *Ibid*, 1895, i. 802.
"Two Cases in which signs of the presence of Intracranial Tumour were followed by Recovery," *Ibid.*, 1897, i. 328.
"On Deep-seated Thoracic Aneurysm," *Clin. J.*, 1896, ix. 106.
"Cases of Hysterical Mutism and Motor Aphasia," *Ibid.*, p. 305.
"On the Use of Oxygen in the Treatment of Anæmia," *Bris. Med.-Chir. J.*, 1896, xiv. 232.
"Case of Acute Miliary Tuberculosis," *Ibid.*, 1897, xv. 148.
"Changes in the Spinal Cord in two cases of Pernicious Anæmia," *Brit. M. J.*, 1897, ii. 325.
(With C. A. Morton) "Operation for Abscess of the Lung due to Localised Necrosis," *Ibid.*, p. 800.
"Two Cases of Congenital Syphilitic Cirrhosis of the Liver in Infants," *Am. J. M. Sc.*, 1898, cxv. 413.

"Case of Cancer of the Pylorus presenting some unusual features,' *Lancet*, 1898, ii. 866.

"Clinical Study of the Gastric Juice," *Brit. M. J.*, 1898, ii. 1863.

"Treatment of Enteric Fever," *Bris. Med.-Chir. J.*, 1898, xvi. 15.

"On the Temperature in Cases of Apoplexy," *Ibid.*, 1899, xvii. 97.

"Epileptic Attacks preceded by Subjective Auditory and Taste Sensations," *Lancet*, 1900, i. 1119.

"Case of Ulcerative Endocarditis treated by Anti-streptococcic Serum," *Ibid.*, 1900, ii. 168.

"Case of Varicose Aneurysm of the Aorta communicating with the Pulmonary Artery," *Brit. M. J.*, 1900, ii. 1701.

(With R. G. P. Lansdown) "Case of Sarcoma of the Brain removed by Operation," *Ibid.*, 1901, i. 879.

"Two Cases of the Sporadic Form of Epidemic Cerebro-spinal Meningitis," *Bris. Med.-Chir. J.*, 1900, xviii. 128.

(With C. A. Morton) "Case of Abscess in the Left Lobe of the Cerebellum successfully Evacuated," *Ibid.*, 1901, xix. 112.

"Two Cases of Localised Necrosis of the Lung," *Ibid.*, 1902, xx. 122.

"Nystagmus," Quain's *Dictionary of Medicine*, 3rd Ed., 1902, p. 1103.

"A Case of Banti's Disease, *Bris. Med.-Chir. J.*, 1903, xxi. 14.

"Treatment of Sciatica," *Lancet*, 1903, ii. 1083.

"Case of Tuberculous Ulceration of the Intestines," *Ibid.*, 1904, ii. 147.

'On the Relation of the Argyll-Robertson Phenomenon to Syphilis," *Brit. M. J.*, 1903, ii. 1634.

"Case of Cured Hydrocephalus," *Brit. J. Child. Dis.*, 1904, i. 503.

"Spinal Cord Degenerations in Anæmia," *Brain*, 1904, xxviii. 441.

"On some Symptoms of Cerebral Tumours," *Bris. Med.-Chir. J.*, 1905. xxiii. 97.

"Presidential Address on the Relation of Medicine to the Natural Sciences" (Bath and Bristol Branch B.M.A.), *Ibid.*, p. 193.

"Two Cases of Myasthenia Gravis," *Ibid.*, p. 308.

*Hysteria and Neurasthenia*. London. John Lane. 1905.

"Cases Illustrating the More Unusual Complications of Pneumonia," *Bris. Med.-Chir. J.*, 1907, xxv. 89, 108.

"Treatment of Graves's Disease," *Ibid.*, p. 201.

"Lymphadenoma Treated by X-Rays," *Brit. M. J.*, 1907, ii. 1137; *Arch. Roentgen Ray*, 1908, xiii. 290.

"Clinical Lecture on Cases of Paraplegia," *Ibid.*, 1908, ii. 699.

"Case of Acute Lymphadenoma," *J. Path. and Bacteriol.*, 1908, xiii. 92.

(With H. J. Mackay) "Case of Hœmorrhagic Myelitis," *Brain*, 1908, xxxi. 514.

"The Renal Changes in a Case of Hæmorrhage into the Pons, with consequent High Blood-pressure," *Bris. Med.-Chir. J.*, 1908, xxvi. 230.

"Recent Work on Leukæmia and some Allied Affections," *Ibid.*, p. 289.

"Precautions to be observed in administration of Milk in Disease," *Ibid.*, 1909, xxvii. 57.

(With E. W. H. Groves) "Remarks on Syringomyelia occurring in Brother and Sister," *Brit. M. J.*, 1909, ii. 737.

"Recurrent Motor Paralysis in Migraine," *Ibid.*, 1910, i. 1534.

"Recurrent Paralysis," Allbutt's *System of Medicine*, 1910, viii. 604.

"Leukæmia Treated by X-Rays," *Bris. Med.-Chir. J.*, 1910, xxviii. 208.

"The Long-Fox Lecture on Cerebrospinal Syphilis," *Ibid.*, 1911, xxix. 1.

"Argyll-Robertson Sign in Cerebral and Spinal Syphilis, *Brit. M. J.*, 1911, i. 296.

"Some Features of Aortic Regurgitation in Young Subjects," *Ibid.*, p. 1364.

"Tumour of the anterior part of the Corpus Callosum extending into the Frontal Lobe," *Ibid.*, 1912, ii. 1447.

"Removal of Extra-Medullary Tumour of Cervical Cord," *Ibid.*, p. 175.

"Case of General Infection by the Influenza Bacillus," *Lancet*, 1912, i. 1465.

"Chronic Interstitial Pancreatitis of Uncertain Origin," *Bris. Med.-Chir. J.*, 1912, xxx. 97.

"Case of Acute Septicæmia due to B. Pyocyaneus," *Ibid.*, 1913, xxxi. 31; and *M. Press and Circ.*, 1913, xcv. 492.

(With C. A. Morton) "Removal of Intrathecal Tumour from Lumbar Region of Spinal Cord," *Brit. M. J.*, 1913, i. 932.

"Diseases of the Lungs associated with the presence of Friedlander's Bacillus," *Bris. Med.-Chir. J.*, 1914, xxxii. 1.

"On Toxic Polyneuritis of the Motor Type," *Ibid.*, 1915, xxxiii. 9.

(With R. G. P. Lansdown) "Intra-Medullary Tumour of the Spinal Cord," *Brit. M. J.*, 1914, i. 1009.

(With J. O. Symes) "Small Outbreak of Epidemic Cerebrospinal Meningitis," *Ibid.*, p. 1286.

"Pathological Changes in Acute Leukæmia from prolonged use of X-Rays," *Proc. Roy Soc Med.*, 1913, vii. Med. Sec., 205.

"The Bradshaw Lecture on Nervous Affections of the Sixth and Seventh Decades of Life," *Lancet*, 1915, ii. 1017, 1069.

"Gunshot Wounds of Peripheral Nerves," *Bris. Med.-Chir. J.*, 1917, xxxv. 61.

"Trench Nephritis," *Brit. M. J.*, 1917, ii. 239.

## Figures and Tables

**Figure f1:**